# Personalized surveillance and aftercare for non-metastasized breast cancer: the NABOR study protocol of a multiple interrupted time series design

**DOI:** 10.1186/s12885-023-11504-y

**Published:** 2023-11-14

**Authors:** A. Klaassen-Dekker, C. H. C. Drossaert, M. C. Van Maaren, A. E. Van Leeuwen-Stok, V. P. Retel, J. C. Korevaar, S. Siesling, on behalf of the NABOR project group, B. Knottnerus, C. Guerrero-Paez, J. Burgers, A. Zeillemaker, M. J. Vrancken, M. Van Hezewijk, E. Siemerink, A. Honkoop, J. Veltman, R. Mann, J. Wiegersma, S. Claassen, M. Van der Lee, N. Van Uden

**Affiliations:** 1 Health Technology and Services Research Department, Technical Medical Centre, University of Twente, Enschede, The Netherlands; 2 Department of Research and Development, Netherlands Comprehensive Cancer Organisation (IKNL), Utrecht, The Netherlands; 3 Health & Technology Department, University of Twente, Enschede, The Netherlands; 4 Dutch Breast Cancer Research Group, BOOG Study Center, Utrecht, The Netherlands; 5 Division of Psychosocial Research and Epidemiology, Netherlands Cancer Institute-Antoni Van Leeuwenhoek Hospital, Amsterdam, The Netherlands; 6 Erasmus School of Health Policy and Management, Erasmus University Rotterdam, Rotterdam, The Netherlands; 7 Faculty of Health, Nutrition & Sport, The Hague University of Applied Sciences, The Hague, The Netherlands; 8 Netherlands Institute for Health Services Research (NIVEL), Utrecht, the Netherlands

**Keywords:** Personalized follow-up, Breast cancer, Surveillance, Aftercare, Risk of recurrence, Decision-making

## Abstract

**Background:**

Follow-up of curatively treated primary breast cancer patients consists of surveillance and aftercare and is currently mostly the same for all patients. A more personalized approach, based on patients’ individual risk of recurrence and personal needs and preferences, may reduce patient burden and reduce (healthcare) costs. The NABOR study will examine the (cost-)effectiveness of personalized surveillance (PSP) and personalized aftercare plans (PAP) on patient-reported cancer worry, self-rated and overall quality of life and (cost-)effectiveness.

**Methods:**

A prospective multicenter multiple interrupted time series (MITs) design is being used. In this design, 10 participating hospitals will be observed for a period of eighteen months, while they -stepwise- will transit from care as usual to PSPs and PAPs. The PSP contains decisions on the surveillance trajectory based on individual risks and needs, assessed with the ‘Breast Cancer Surveillance Decision Aid’ including the INFLUENCE prediction tool. The PAP contains decisions on the aftercare trajectory based on individual needs and preferences and available care resources, which decision-making is supported by a patient decision aid. Patients are non-metastasized female primary breast cancer patients (*N* = 1040) who are curatively treated and start follow-up care. Patient reported outcomes will be measured at five points in time during two years of follow-up care (starting about one year after treatment and every six months thereafter). In addition, data on diagnostics and hospital visits from patients’ Electronical Health Records (EHR) will be gathered. Primary outcomes are patient-reported cancer worry (Cancer Worry Scale) and overall quality of life (as assessed with EQ-VAS score). Secondary outcomes include health care costs and resource use, health-related quality of life (as measured with EQ5D-5L/SF-12/EORTC-QLQ-C30), risk perception, shared decision-making, patient satisfaction, societal participation, and cost-effectiveness. Next, the uptake and appreciation of personalized plans and patients’ experiences of their decision-making process will be evaluated.

**Discussion:**

This study will contribute to insight in the (cost-)effectiveness of personalized follow-up care and contributes to development of uniform evidence-based guidelines, stimulating sustainable implementation of personalized surveillance and aftercare plans.

**Trial registration:**

Study sponsor: ZonMw. Retrospectively registered at ClinicalTrials.gov (2023), ID: NCT05975437.

## Introduction

Each year in the Netherlands, about 15.000 non-metastasized female breast cancer patients start with their follow-up [1]. This growing number of breast cancer survivors increases the demand on follow-up care, which consists of surveillance and aftercare. Surveillance focuses on early detection of locoregional recurrences (LRR) or second primary breast cancers (SPBC) using mammograms and physical examination. Aftercare focuses on prevention, early recognition and treatment of possible (late) physical or psychological effects of breast cancer and its treatment [2].

Currently, most Dutch hospitals organize surveillance as ‘one-size fits all’ [2], following the national guideline by offering an annual mammogram and physical examination. However, the benefits of these pre-scheduled surveillance visits are limited and may differ by patient. The risk of recurrence is low [3], depends on patient-, tumor- and treatment-related characteristics and varies over time [4–6]. For women with a low risk, annual mammograms may be too frequent. Especially since studies have shown that more surveillance does not guarantee less severe recurrences [7, 8] and about half of the recurrences are detected outside of scheduled visits by patients themselves [9]. Intense surveillance has not only limited benefits, but also places a burden on patients and healthcare. For patients, surveillance may provoke distress and cancer worry, which is a very prevalent concern among cancer survivors and can negatively impact quality of life [10]. Even more, it may contribute to unrealistically high expectations of the benefits of surveillance and risk perceptions, which hinder the patient’s transition to normal life [11]. Concerning healthcare, intense surveillance may lead to unnecessary costs and use of limited care resources, especially when provided even more intensively than guidelines recommend [7, 12]. Taken together, current organization of surveillance neglects patients’ prognoses and preferences, which may cause unnecessary burden and less efficient care.

Compared to surveillance, the organization of aftercare is less structured, with more variation between hospitals [13], probably because guidelines are not specific about the schemes of consultation and information provision. Examples of variation between hospitals are the intensity, involvement of type of professionals, use of tools for needs assessment and information provision [13]. Although several tools exist to detect needs for psychosocial support or to estimate individual risks of late health complaints [14], structural application of these tools seems limited [7, 13]. Even more, patients’ most reported need for support for cancer worry seems often not met [15]. Since such unmet needs may lower overall quality of life [16], it is important to regularly assess needs and stimulate patients’ self-management in the prevention and treatment of their complaints [17]. Because of these reasons and the diversity of patients’ prognoses, symptoms, needs and preferences, personalization of surveillance and aftercare is recommended [4, 17–21].

However, evidence on the effectiveness of personalisation is lacking, as well as a clear direction on how to personalise follow-up. The review of Van Maaren et al. [22] shows that existing interventions to personalize follow-up are scarce (especially on surveillance), vary widely, are not structurally embedded in clinical practice and do not provide clear evidence on its effectiveness. Even more, these interventions are called ‘personalized’ but target different aspects of follow-up in different ways, probably because a definition of personalized surveillance and aftercare is lacking [22].

In this study, personalized surveillance is defined as a surveillance trajectory based on individual risk estimations of cancer recurrence and patients’ preferences, of which a surveillance plan (e.g. on intensity, duration and mode of discussing results) is created in shared decision-making [17, 22]. To estimate these individual risks and to support decision-making on surveillance, a risk prediction tool INFLUENCE [23] and Decision Aid, the ‘Breast Cancer Surveillance Decision Aid’ [24], has been developed in the SHOUT-BC study [25]. This Decision Aid supports shared decision-making as it provides individual risk information, as indicated by INFLUENCE, and assesses patients’ needs. The use and appreciation of these tools are currently being evaluated in the SHOUT-BC study, of which preliminary results show that patients report a lower risk perception, choose less intense surveillance and experience more shared decision-making (Ankersmid et al., personal communication).

Also concerning aftercare, patients should be involved in decision-making, to stimulate their self-management of complaints and to make sure that their needs are discussed [17]. This requires patients’ insight in their needs and available options of support. Therefore, in this study personalized aftercare is defined by the following elements: 1) assessments of patients’ needs, 2) information provision on potential side effects of cancer (treatment) and available care resources, and 3) creation of a personalised aftercare plan which reflects decisions on the aftercare trajectory [22]. These elements may give patients tools to improve their quality of life and return to their day to day activities.

In general, personalization of surveillance and aftercare may facilitate more efficient and valuable care, which may promote patients’ quality of life and satisfaction with care, reduce their cancer worries and unnecessary costs and burdens. To provide evidence on its effectiveness, a large multicentre prospective study is needed. Therefore, this study investigates the (cost-)effectiveness of a Personalised Surveillance Plan (PSP) and Personalized Aftercare Plan (PAP) on cancer worries and quality of life. Next, we will investigate the effects on risk perception, shared decision-making, patient satisfaction and needs for support, mental adjustment to cancer, societal participation and costs (including health care and resource use, work productivity). Also several tools will be used to support implementation of a PSP and PAP. For the PSP, this study will built upon the SHOUT-BC study [25] by using the (improved) Breast Cancer Surveillance Decision Aid including an updated version of INFLUENCE. For the PAP, we will develop a new Aftercare Decision Aid in this study. This Decision Aid will support shared decision-making as it will provide insight in patients’ needs and wishes and inform them of possible effects of breast cancer and available care resources. Finally, this study will evaluate the implementation, uptake and appreciation of these tools. Results of this study will support revision and implementation of the guidelines on personalised surveillance and aftercare, supporting more personalized care.

## Methods

### Design

**Fig. 1:**
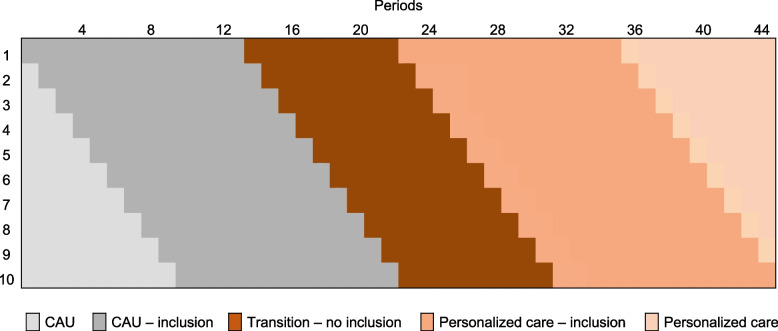
Study design: stepwise transition from care as usual to personalized care in ten hospitals. One period is three weeks

We use a prospective multicenter multiple Interrupted Time Series design (mITS) to compare the effectiveness of personalized follow-up (surveillance and aftercare) with current follow-up, taken the possible impact of changes over time into account. Through a continuous sequence of observations (i.e. data from questionnaires and registries), taken repeatedly at equal time intervals, underlying trends can be established on outcomes of interest. First, hospitals provide CAU for at least 12 periods of 3 weeks (approximately nine months) while including participants for this study (Fig. 1). Next, they enter stepwise the transition period of 9 periods of 3 weeks (approximately six months) in which HCPs receive training on using personalized surveillance (PSP) and aftercare plans (PAP) and supporting tools. From the transition period onwards, hospitals provide personalized care for all their patients who start with their follow-up (Fig. 1). During the transition period, no patients will be included in this study, since not all of them would be provided with both a PSP and PAP. After the transition period, hospitals will include participants for this study during the following 12 periods in which all patients will be provided with personalized care. It will take about nine months until all participating hospitals have entered the transition period and provide personalized care, which they will continue to provide after the end of inclusion of this study.

### Study setting

Fifteen independent Dutch hospitals previously expressed their interest to participate in this study. From these, we selected ten hospitals which are geographically spread across the Netherlands. We selected based on the following inclusion criteria: no current provision of personalized follow-up (e.g. by providing surveillance based on individual risks), no significant barriers to participation (e.g. upcoming mergers, lack of manpower or motivation among the team) and having a sufficient amount of patients for inclusion. The latter was based on preliminary analysis of data from the Netherlands Cancer Registry and related to our sample size calculations. After visiting all potential participating hospitals to discuss feasibility of participation, we approved ten hospitals. A list of participating hospitals can be obtained at ClinicalTrials.gov (NCT05975437).

### Participants and procedures

The study population consists of all new female patients who were curatively treated for non-metastasized primary breast cancer and start follow-up care. Inclusion criteria are: 1) female, 2) aged 40 years or older (because of higher risk on recurrence), 3) facing the decision for the organization of post-treatment surveillance and aftercare, 4) being curatively treated including breast surgery, for invasive non-metastasized breast cancer and 5) able to understand the Dutch language in speech and writing. Exclusion applies to 1) bilateral breast cancers, 2) BRCA1/2 or CHEK2 carriers, 3) having an indication for MRI and 4) participation in another study that requires fixed scheduled follow-up consultations and/or imaging. Each period of three weeks, the first eight patients who meet the inclusion criteria and come to the hospital for follow-up consultation on the occasion of their first post-treatment surveillance with imaging (about one year after surgery) will be invited by their HCP. During this consultation, patients will be informed and asked to participate in this study. When interested, patients receive a patient information letter about the study and asked for written informed consent. Presumably, half of the informed patients is willing to participate, resulting in the required inclusion of four participants per period. Patients who decline participation in the NABOR study will still receive the same form of care (CAU or personalized care), depending on whether the hospital is in the pre- or post transition phase.

### Treatment of subjects

#### Care as usual (CAU)

This study compares the effectiveness of personalized care to CAU, which is generally non-personalized. During the phase of CAU, all patients receive the usual type of follow-up care. For the majority of hospitals this includes annual mammography and physical examination combined with discussion of the results of the mammogram and aftercare consultations.

#### Personalized Surveillance Plan (PSP)

Around the first surveillance mammogram (i.e. one year after end of treatment), the personalized surveillance plan (PSP) is generated by means of the PSP decision aid, called the ‘Breast Cancer Surveillance Decision Aid’ [24]. This Aid incorporates the INFLUENCE tool 3.0, which is a sequel of the INFLUENCE tool 2.0 [23] and will be developed during this project. Compared to INFLUENCE 2.0, INFLUENCE 3.0 will additionally include patients treated with neoadjuvant therapy a broader population than INFLUENCE 2.0 (including patients treated with neoadjuvant therapy) and will include a more recent population in order to provide more contemporary risk estimates that are applicable in a broader population. During an outpatient clinic visit, around the first surveillance mammogram (i.e. approximately one year after the diagnosis), the INFLUENCE 3.0 prediction tool, as part of the PSP decision aid, is completed by the HCP (i.e. surgical oncologist or nurse specialist) and patient together by filling in data on patient, tumour and treatment characteristics. The estimated personal risk will be explained to the patient and summarized on a leaflet that also outlines the options possible for the patient (e.g. annual mammogram or less frequent, duration of follow-up, how to deliver the result from the mammogram). The patient receives a personal account with which she can, at home, complete the PSP decision aid that provides information about different surveillance options and a value clarification exercise to help the patient to get insight in her personal needs and preferences. This helps the patient to consider the pros and cons of the different options (annual mammogram, or less often). A summary sheet based on patients’ answers, combined with the result of the first surveillance mammogram, is used in the next consultation to make shared decisions on a personalized surveillance plan (PSP).

#### Personalized Aftercare Plan (PAP)

To support creation of this PAP, an aftercare decision aid will be used, which assesses patients’ needs, offers information and provides a summary of patients’ needs and preferences regarding the aftercare trajectory. The content of this aftercare decision aid will be developed in five cocreation sessions with a multidisciplinary team of researchers, patient representatives and care providers (i.e. oncologists, nurse specialists, general practitioners, etc.). First, needs assessment studies among patients and care providers will be conducted, which results serve as input for the content of the aftercare decision aid. This content will be critically revised by the team and rewritten to B1 language level (Common European Framework of Reference for Languages). Usability will be tested, consisting of think-aloud sessions with patients and interviews by telephone among health care professionals.

During aftercare consultation(s) in the first year after the end of patients’ treatment, the HCP (i.e. nurse or nurse specialist) will introduce the aftercare decision aid to the patient. Next, patients access the online aftercare decision aid to complete a needs assessment and receive information about possible effects of breast cancer, available options and choices that she has concerning her aftercare trajectory and available resources for help and support. Patients can weigh options and fill in preferences and considerations. Once patients have completed the aftercare decision aid, a summary sheet will automatically be created, containing an overview of patient-reported needs, preferences and considerations, which can be used as a base for final decision-making on the PAP in a consultation with their care provider. Examples of decisions are the frequency of consultations, type of caregiver and the mode of following contact moments (face to face, video consultation, via telephone or email), in accordance with available resources of each hospital and regional care network. These decisions will compose the PAP, which will most likely include the following elements: a diagnosis and treatment summary; decisions on organisation of aftercare (e.g. further support or referrals, mode of contact, involved care providers) and signals to seek care for; contact details and links to further information (e.g. about possible complaints and resources for help and support). Since patients’ needs, preferences and situations may vary over time, the care provider can introduce the aftercare decision aid multiple times during the aftercare trajectory and patients can refer to the aftercare decision aid when desired. Also the PAP might be re-evaluated and adapted during the aftercare trajectory, depending on patients’ needs and arranged contact frequencies.

#### Data collection and outcome measures

**Fig. 2:**
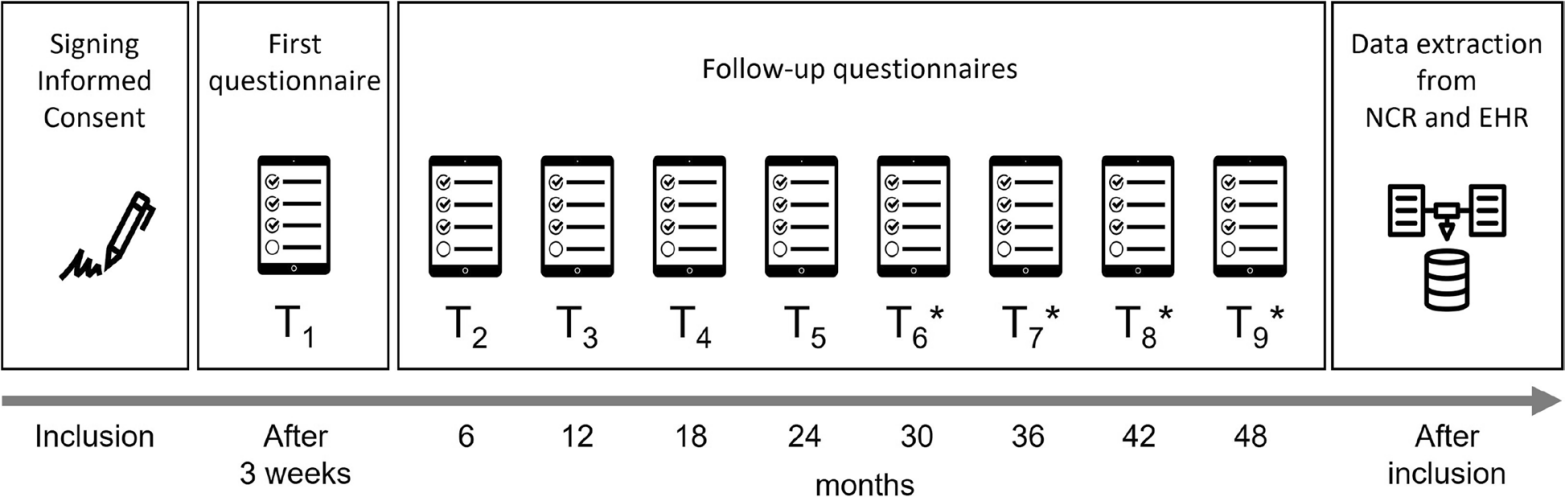
Participant timeline. The procedure for patients included during CAU and personalized care are the same. Moment of first measurement is one year after treatment is ended, around the first mammogram. NCR = Netherlands Cancer Registry; EHR = Electronic Health Records. *only applies to a subsample of the first 50 patients included during care as usual (CAU) and the first 50 patients included during personalized care

Data will consist of questionnaires and registered data collected via the Netherlands Cancer Registry (NCR) and additional data from the Electronic Health Records (EHR) (Fig. 2).

#### Questionnaires

An overview of all questionnaires and used instruments is provided in Table 1. A baseline questionnaire (T1) will be sent to patients, via e-mail or post, after informed consent is signed (i.e. approximately one year after initial treatment). Subsequently, patients will receive five follow-up questionnaires during two years: after six (T2), twelve (T3), eighteen (T4) and twenty-four (T5) months. For a subsample of patients (the first 50 patients included in the current care phase and the first 50 patients included from the personalized care phase) we will collect for an additional two years the health care consumption. This longer follow-up enables estimation of cost-effectiveness over a 5-year period, which is currently the standard duration of follow-up in hospitals. Therefore, this subsample receives four extra questionnaires: after thirty (T6), thirty-six (T7), forty-two (T8) and forty-eight (T9) months. The time it takes to complete the questionnaires differs per measurement moment. The T1 questionnaire takes about 45 min to complete, the T3 and T5 questionnaires take around 30 min and the T2 and T4 (and T6, T7, T8 and T9) questionnaires take around 15 min to complete.

**Table 1:** Detailed overview of included measures in the patient questionnaires

Measure	Description	Scoring range	T1	T2	T3	T4	T5	T6	T7	T8	T9
Cancer worry; CWS [26] (primary outcome)	6-item, 4-point scale measures concerns about cancer recurrence and the impact of these concerns on daily functioning	Range 6–24, higher scores indicate greater worrying	X		X		X				
Self-rated quality of life; VAS score [27] (primary outcome)	1 item, measures patients’ health-related quality of life	Range 0–100, higher scores indicate greater health-related quality of life	X		X		X				
Quality of life (EQ-5D-5L) [27]	5 items, 5-point scale measures patients’ health-related quality of life		X		X		X				
Short-Form health index (SF-12) [39]	12 items with 2–6 response options on quality of life	Mental and physical component score based on the US population scoring system, higher scores indicate greater quality of life	X		X		X				
Physical symptoms scale of the EORTC-QLQ-C30 [40]	13 items, 4-point scale measure physical symptoms		X		X		X				
Healthcare consumption; selection of iMTA Medical Consumption Questionnaire (iMCQ) [41]	6 items, measure frequency of visits to health care practitioners outside the hospital and the extent to which others needed to join the patient in going to these visits, and the use of home care and medication		X	X	X	X	X	X	X	X	X
Work productivity; selection of iMTA Productivity Cost Questionnaire (iPCQ) [33]	3 items measure productivity losses of paid work due to absence from work, reduced productivity while at work and productivity losses related to unpaid work		X	X	X	X	X	X	X	X	X
Perceived risk of recurrence [25]	3 items measure patients’ perceived absolute risk, perceived comparative risk (compared to the average risk in women who have had breast cancer) and perceived course of the risk over time		X		X		X				
Patient satisfaction; selection of CQ-breast index [42]	27 items, measure clarity of received information, the accessibility and form of treatment of health care practitioners and satisfaction with received care		X		X		X				
Patients’ need for support for fear of cancer recurrence (selection of questionnaire used by Luigjes-Huizer and colleagues [15])	7 items representing seven types of support which can be offered in the hospital. Assesses per type of support whether patients needed and received the support, and, if so, whether it helped		X		X		X				
Shared Decision-Making Questionnaire (SDM-Q-9) [43]	9 item, 6-point scale measures patients’ perceived level of involvement in decision-making	Range 0–45, higher scores indicate a greater level of perceived involvement in decision-making	X								
Patients’ role in decision-making; Control Preference Scale (CPS) [44]	2 items with 5 response options to assess the patient’s preferred and perceived degree of control when decisions about treatment are being made		X								
Health literacy; Set of Brief Screening Questions (SBSQ) [45]	3 items, 5-point Likert scale measure health literacy	An average score of ≤ 2 indicates inadequate health literacy, and a score > 2 indicates adequate health literacy	X								
Mental adjustment to cancer; subscales ‘Fighting spirit’ and ‘Helplessness/hopelessness’ of the Mental Adjustment to Cancer scale (MAC) [46]	22 items, 4-point scale measures positive and negative mental adjustment to cancer	Higher scores on Fighting Spirit indicate greater positive mental adjustment, higher scores on Helplessness/hopelessness indicate greater negative mental adjustment to cancer	X								
Social engagement and participation; selection of the Lubben Social Network Scale (LSNS) [47]	3 items measuring social engagement in terms of size, closeness and frequency of contacts of a respondent’s social network with family and friends, with 6 response options on number of engaged family members or friends	Higher scores indicate greater social engagement	X		X		X				

#### Registered data

Patient-, tumor- and treatment-related characteristics and possible recurrence of disease will be collected from the NCR at the end of the study. These characteristics are routinely gathered in the NCR for all Dutch cancer patients. The NCR is hosted by the Netherlands Comprehensive Cancer Organization (IKNL) who has specially trained registrars extracting data directly from the patients files in all hospitals in the Netherlands. Additional relevant data (e.g. data on diagnostics/imaging, breast cancer-related hospital visits) will be retrieved from EHRs.

#### Outcomes

A detailed overview of all used instruments is provided in Table 1. Below a summary of outcome categories. The primary outcome to assess the effectiveness of personalized surveillance (PSP) and aftercare plans (PAP) will be patients’ experienced level of cancer worry and patients’ overall self-rated quality of life (EQ-VAS-score). Secondary outcomes will be: (1) quality of life including patients’ daily functioning and symptoms, (2) cost-effectiveness, by comparing societal costs and effects (i.e. healthcare costs, work productivity), (3) perceived risk of recurrence, (4) satisfaction and experiences with care, (5) patients’ need for support for fear of cancer recurrence, (6) shared-decision-making and (7) coping strategy in adjusting to cancer. Sociodemographic factors will be assessed in the first questionnaire: marital status, education level, work status, health literacy, social network and social engagement. Clinical characteristics will also be collected, namely tumor and treatment characteristics and possible cancer recurrences, which will be obtained from the NCR. Additional relevant data from the EHR, concerning the use of prediction models and patient decision aids, performance of imaging (i.e. conclusion of imaging, breast density, reason for consults) and pathology (i.e. type of biopsies, cytology, histology), detected recurrences and reason of breast cancer-related hospital visits, will be retrieved. This data from the EHR will be used to estimate healthcare costs and effects and to evaluate use of PSP and PAP.

#### Sample size

The sample size was estimated using a user-written script for mITS designs in the Statistical Analysis System (SAS) software program. Since we have two primary parameters, we correct for multiple testing using a Bonferroni correction and therefore set the statistical significance level at alpha = 0.025 (two sided). The effects we wish to measure are a difference of 1.52 on the Cancer Worry Scale (CWS; range 6–24) and a difference of 4.8 on the EQ-VAS score (range 0–100), which is part of the EQ-5D. This decision is based on our aim to detect a small to moderate difference of 0.4 times the standard deviation [24], which was found to be around 3.8 for the CWS [26] and 12 for the EQ-VAS score [27] in previous studies. From a clinical point of view, a difference of 1.52 on the CWS is relevant for our purpose to estimate the effectiveness of personalized follow-up: even a small decrease in cancer worry can lead to improved quality of life [28]. A difference of 4.8 on the VAS score is considered to be clinically relevant as well. We expect a correlation between the first two measurements within one hospital of 80%, and we expect this correlation to drop to 50% when comparing the first with the last measurement. In addition, we assume an intraclass correlation coefficient of 0.15. Taken 25% loss-to-follow up into consideration, each hospital will have to include four patients per period of three weeks in order to detect a difference of 1.52 on the CWS and 4.8 on the VAS score with 84%. The total inclusion time per hospital is 26 periods (78 weeks, or approximately 18 months), which amounts to a number of 104 patients per hospital and a study population of *N* = 1,040.

### Statistical analyses

An overview of the demographic and clinical characteristics will be provided using descriptive statistics. Continuous data will be expressed as a mean with the standard deviation (SD), or the Interquartile range (IQR) where appropriate. Categorical data will be expressed as frequencies (%). All questionnaires will be analysed in accordance with their corresponding manual. Self-composed questions (i.e. perceived risk of recurrence, adjusted questions from the CQ-Breast Index, demographics) will be analysed per item.

To assess the effectiveness of personalized surveillance and aftercare, all outcome parameters will be compared between the current-care and personalized-care groups. We will visualize the time series patterns pre- and post-personalization to assess a possible change in pattern after implementation of the personalization, for each hospital separately and combined. In this way we can also identify any underlying trends, seasonal patterns and outliers.

To test the change in level and slope associated with the personalization and to control for other (confounding and overall trend) effects, we will use segmented regression analyses in which piecewise regression lines will be fitted to each segment of time series, allowing each segment to exhibit different trends [29]. To correct for correlation between repeated measurements we will visually examine residual plots against time, which can additionally be statistically tested using the Durbin-Watson statistic. Autocorrelation will consequently be adjusted for by including the autocorrelation parameter in the segmented regression model [30]. Intention-to-treat analyses are done to estimate the effectiveness of personalized follow-up on the outcomes of the questionnaires. We will use the Bonferroni-Holm correction to adjust for multiple testing, meaning that the smallest two-sided p-value will be compared to 0.025 and if successful, the largest two-sided p-value will be compared to 0.05.

In case of missing data, we will record the percentage of drop-out and missing at each follow-up timepoint. If necessary, we will perform multiple imputation (if the assumptions for this technique are met) in order to ensure accurate analysis. In case some participants do not answer a section of the questionnaires, we will perform sensitivity analyses with and without the group with incomplete answers to detect possible differences in effects. Thereafter, we will perform meta-analyses to evaluate the combined results of all included hospitals. While the interrupted time series is generally considered the methodologically strongest non-randomized design, we will increase validity of its conclusions by confounder adjustment. As the effects are based on within-hospital comparisons, confounders at hospital level are dealt with by design. We will adjust for possible prognostic characteristics at patient level, i.e. patient-, tumour- and treatment-related characteristics (e.g., age, socioeconomic status, tumour and nodal stage, differentiation grade, receptor status, radiotherapy, systemic therapy, targeted therapy). Analyses to investigate effect modification and effect moderation by patient-, tumour- and treatment-related characteristics will be performed as well.

### Cost-effectiveness analysis

A cost-effectiveness and cost-utility analysis will be performed comparing costs and effects of “personalized follow-up care” versus “care as usual”, using a two-year (study-based; based on data from the current study) and lifetime (model-based; based on the study and extrapolations by means of data from literature) time horizon. We will derive information of the impact on personalized follow-up care on cancer worries, QoL, healthcare costs and (shift in) resources. 

Direct healthcare costs based on activities extracted from the EHRs (e.g. number of mammograms, consultations) will be multiplied by costs described in 'Benchmark costs from the Netherlands' or from the ‘Nederlandse Zorgautoriteit’ (NZa) [31]. For the costs of the interventions (decision support tools), an activity-based costing method will be performed for development, use and maintenance of the decision support platforms [32]. Indirect costs that will be taken into consideration are healthcare consumption outside the hospital and health-related productivity losses. Productivity losses will be measured by means of a selection of iMTA Productivity Cost Questionnaire (iPCQ) [33] and calculated by means of the Friction cost method, according to the International Society of Pharmaco-economical Organization and Research (ISPOR) guidelines [34].

The cost-effectiveness will be expressed in incremental costs per patient with a clinically relevant improvement on the CWS as primary outcome of the NABOR study. The cost-utility will be expressed in incremental costs per quality adjusted life years (QALYs) gained, obtained from the EQ-5D-5L (including the VAS) [27]. Long term consequences (after the timelines of this study), e.g. in terms of cancer worries and utilities, will be based on both the study results and literature to extrapolate the patient outcomes for a lifelong time horizon. The cost-effectiveness analysis will be performed by means of a state-of-the-art decision model, according to the guidelines for economic evaluations of the Dutch Zorginstituut (ZIN, [31]), like conducted by De Ligt and colleagues [35]. Uncertainty around the results will be quantified by means of non-parametric bootstrapping and cost-effectiveness acceptability curves, showing the possibility of the personalized follow-up care being whether or not cost-effective for various values of the Dutch society willingness to pay for one QALY [30].

## Discussion

This study supports the implementation of personalized surveillance and aftercare by developing and updating supporting tools and may provide new insights on the (cost-)effectiveness of personalized follow-up care, based on individual risks and needs.

### Risks and challenges

However, we foresee some risks and challenges that need a strategy. The first risk to encounter would be insufficient numbers of inclusion, which we will try to prevent by clear instructions, real-time monitoring of progress in each hospital and early assessment of barriers and facilitators. In the worst case a hospital turns out to be unable to achieve the required numbers of inclusion, we may approach other hospitals that expressed to be interested in participation. Second, structurally assessing patients needs before making decisions on personalized aftercare may identify more needs that were otherwise unknown, potentially resulting in more use of help and support. Although this may result in increasing costs at first, earlier treatment of symptoms may also prevent health effects later on. Next, it may be challenging to make shared decisions on the intensity of aftercare consultations within the limited capacity of the health care system. Therefore, management of patients’ expectations by communicating aftercare options and providing a clear explanation on what signals call for contacting a care provider is important. Another challenge is learned from the SHOUT-BC study, which already implemented the ‘Breast Cancer Surveillance Decision Aid’ and observed that care providers often not structurally explain available options and choices in surveillance and limitedly address patients’ considerations. Since the effectiveness of personalized surveillance depends on whether patients are well informed and involved in shared decision-making, we will provide early and intensive training of care providers, as well as evaluate the decision-making process post-implementation. Furthermore, it is known that patients and HCPs may be reluctant to opt for less intense surveillance, for instance because of patients’ fear of recurrence [12, 19, 36] or HCPs’ hesitance to rely on the risk information of the INFLUENCE nomogram. To promote use of risk information and open discussion of all surveillance options, the existing base of evidence [6, 7, 37, 38] regarding less intense surveillance schemes and use of risk information will be communicated before and during the transition period. At the end of the study, we can evaluate the eligibility of concerns regarding less intensive surveillance, since we also compare data on detected recurrences during patients’ participation with their individual risks, surveillance schemes and compare differences between patients who received CAU or a PSP. By collecting information on aftercare and surveillance consultations from the EHR, we can also evaluate whether a PAP and PSP is created and to what extent the actual provided care is in alignment with the decisions in the PSP and PAP. Lastly, the effectiveness of using a PSP and PAP may differ per patient and per hospital. Patients may benefit from the PSP and PAP differently, probably dependent on their tumour- and treatment-related characteristics, but also personal characteristics like their capability to understand and use the tools and ability to recognise and apply needs in decision-making. Also hospitals may differ because of differences in patient population, in involvement of care providers and in regional care networks. Therefore, our analyses will be based on within-hospital comparisons and adjusted for possible prognostic factors at patient level. Also, the heterogeneity between hospitals can contribute to complementary outcomes that are generalisable to daily clinical practice.

### Dissemination

Since the beginning of the grant application for this study, we have collaborated with patient representatives and patient associates. This collaboration will continue throughout the study. We will share our study findings through peer-reviewed journals, (inter)national conferences, workshops, webinars, and newsletters and social media. Moreover, the results will be presented in lay language for the general public and presented on websites, such as the one from IKNL and the Dutch Breast Cancer Association. These lay publications will be prepared together with the Dutch Breast Cancer Association. More broadly, all findings will be actively shared with interested healthcare professionals and other interested parties in the Netherlands. The results of this study will also be shared with the guideline working group of NABON (the multidisciplinary National Breast cancer working group) and the Federatie Medisch Specialisten. Thereby, this study contributes to developing evidence-based guidelines, implementing recommendations on personalized surveillance and aftercare plans in alignment with this study. After the study, hospitals can access the INFLUENCE 3.0 model online via Evidencio and use the decision aids under subscription with the development company ZorgKeuzeLab.

Altogether, insights of this study will help to fill the current gaps in evidence on the effectiveness of personalisation and implementation of a Personalized Surveillance Plans and Personalized Aftercare Plans may result in less cancer worries and unnecessary burden to the patient, increase quality of life and decrease the costs of follow-up. In the future, the results of this project, i.e. the developed decision aids, can also be used for personalization of survivorship care for other cancer survivors, e.g. colon cancer survivors.

### Trial status

At the time of submission this trial is currently recruiting participants.

## Data Availability

Not applicable.

## Abbreviations

BRCA1/2: Breast cancer gene mutations
CAU: Care as usual
CHEK2: Mutation of the tumor suppressor gene
CPS: Control Preference Scale
CWS: Cancer Worry Scale
HER: Electronical Health Records
EORTC-QLQ-C30: European Organization for Research and Treatment for Cancer Quality of Life Questionnaire
EQ-VAS score: Score on the visual analogue scale of the EQ-5D quality of life questionnaire
EQ-5D-5L: Quality of life questionnaire
HCP: Health care practitioner
iMCQ: IMTA Medical Consumption Questionnaire
iPCQ: IMTA Productivity Cost Questionnaire
ISPOR: International Society of Pharmaco-economical Organization and Research
LRR: Locoregional recurrence
LSNS: Lubben Social Network Scale
MAC: Mental Adjustment to Cancer scale
MITs: Multiple interrupted time series
MRI: Magnetic Resonance Imaging
NCR: Netherlands Cancer Registry
Nza: Nederlandse Zorgautoriteit
SBSQ: Set of Brief Screening Questions
SDM-Q-9: Shared Decision-Making Questionnaire
SF-12: Short-Form health index
PAP: Personalized aftercare plans
PSP: Personalized surveillance plans
PROMS: Patient-reported outcome measures
QALY: Quality adjusted life year
QoL: Quality of life
SPBC: Second primary breast cancer
ZIN: Zorginstituut Nederland
